# Cardiovascular disease in adolescent and young adult survivors of non-Hodgkin lymphoma in Korea

**DOI:** 10.1007/s11764-025-01816-2

**Published:** 2025-05-16

**Authors:** Keun Hye Jeon, Danbee Kang, Dong Wook Shin, Hyunsoo Kim, Hea Lim Choi, Sang Eun Yoon, Hyunkyung Park, Su-Min Jeong, Juhee Cho

**Affiliations:** 1 Department of Family Medicine, CHA Gumi Medical Center, CHA University School of Medicine, Gumi, Republic of Korea; 2 Department of Clinical Research Design and Evaluation, Samsung Advanced Institute for Health Science and Technology, Sungkyunkwan University School of Medicine, Seoul, Republic of Korea; 3 Center for Clinical Epidemiology, Samsung Medical Center, Sungkyunkwan University School of Medicine, Seoul, Republic of Korea; 4 Department of Family Medicine/Supportive Care Center, Samsung Medical Center, Seoul, Republic of Korea; 5 Department of Family Medicine/Executive Healthcare Clinic, Severance Hospital, Yonsei University College of Medicine, Seoul, South Korea; 6 Division of Hematology-Oncology, Department of Medicine, Samsung Medical Center, Sungkyunkwan University School of Medicine, Seoul, Republic of Korea; 7 Department of Hematology, Asan Medical Center, University of Ulsan College of Medicine, Seoul, Republic of Korea; 8 Department of Medicine, Seoul National University College of Medicine, 1, Gwanak-Ro, Gwanak-Gu, Seoul, 08826 Republic of Korea; 9 Department of Family Medicine, Seoul National University Hospital, Seoul, Republic of Korea

**Keywords:** Non-Hodgkin lymphoma, Adolescents and young adults survivors, Cardiovascular disease

## Abstract

**Purpose:**

Long-term cardiovascular risk in adolescent and young adult (AYA) survivors of non-Hodgkin lymphoma (NHL) remains insufficiently characterized. This retrospective cohort study investigated the incidence of cardiovascular disease (CVD) among AYA survivors of NHL.

**Methods:**

We identified 4553 individuals aged 15–39 years diagnosed with NHL between 2006 and 2019 using the Korean National Health Insurance System database. A control group of 13,659 individuals without a history of cancer or CVD was selected using 1:3 matching based on age, sex, and residential area. The primary outcomes were major adverse cardiovascular events such as myocardial infarction, cardiomyopathy, heart failure, ischemic stroke, and hemorrhagic stroke. Hazard ratios (HRs) and 95% confidence intervals (CIs) were estimated using Cox proportional hazard models.

**Results:**

The mean (SD) age of the participants was 29.5 (6.8) years, and 59% were male. Over a median follow-up of 6.5 years, survivors of NHL had significantly higher risks of cardiomyopathy (HR 6.77; 95% CI 3.44–13.33), heart failure (HR 4.90; 95% CI 3.45–6.97), and hemorrhagic stroke (HR 3.14; 95% CI 1.75–5.65), compared to controls. In the subgroup analyses stratified by treatment modality, the highest risks were observed among patients who underwent hematopoietic stem cell transplantation, which involved high-dose chemotherapy with or without radiotherapy. The risk of myocardial infarction and ischemic stroke did not increase significantly.

**Conclusion:**

AYA survivors of NHL had a significantly higher risk of CVD, including cardiomyopathy, heart failure, and hemorrhagic stroke, than the general population.

**Implications for Cancer Survivors:**

Long-term cardiovascular surveillance is essential for AYA survivors of NHL, particularly those receiving intensive treatment.

**Supplementary Information:**

The online version contains supplementary material available at https://doi.org/10.1007/s11764-025-01816-2.

## Introduction

An estimated 1.2 million adolescents and young adults (AYA) aged 15–39 years were diagnosed with cancer globally in 2020 [1]. With advances in cancer treatments, the 5-year relative survival rate of AYA patients with cancer has increased over the past few decades [2]. In Korea, the 5-year relative survival rate of AYA patients with cancer has also improved, rising from 58.4 (1993–1995) to 81.4% (2012–2016) [3]. Consequently, more young people are now long-term cancer survivors; however, they face significant long-term health impacts, often dealing with chronic conditions related to cancer treatment [4–6]. Cardiovascular disease (CVD), in particular, is one of the most debilitating late effects encountered by childhood and AYA cancer survivors [7, 8] and is a leading cause of non-cancer mortality [6]. However, data on this population are limited, with much of the research being extrapolated from studies on childhood cancer survivors, detailing the risk of chronic comorbidities [9]. Considering the important social and biological differences among AYA cancer survivors [10], research exploring the relationships among cancer, treatment, patient-related risk factors, and cardiovascular outcomes in this population is warranted.

Non-Hodgkin lymphoma (NHL) is one of the most common malignancies in AYA [11].Notably, NHL in the South Korean AYA population showed the second greatest increase in incidence between 1993 and 2016, with an annual percentage change of 13.4% [3]. Although the late effects of childhood NHL have been demonstrated in several studies of childhood cancer survivors [4, 5, 12], little is known about long-term complications among AYA survivors [13]. Most studies investigating the late effects of AYA survivors of NHL included them as a subset of a larger cohort. In these studies, AYA survivors of NHL had a more than 1.4-fold increased risk of developing CVD compared to the general population [14–16] or siblings [17] without cancer. Only a few studies have described the specific risks for subgroups of AYA survivors of NHL categorized by demographic factors (such as age at diagnosis [16, 18]) and clinical characteristics (such as treatment modalities [18, 19]). Notably, all of these studies were conducted in Western populations, with no published data available for Asian populations.

Therefore, we aimed to assess the various cardiovascular outcomes after NHL treatment in AYA survivors using a nationwide population-based dataset. In addition, we investigated risk factors independently associated with a higher incidence of cardiovascular outcomes.

## Methods

### Data source and study setting

We conducted a retrospective population-based cohort study using data from the Korean National Health Insurance Service (K-NHIS) database. Data on the use of medical facilities and prescription records using the International Statistical Classification of Diseases and Related Health Problems, Tenth Revision (ICD-10) diagnosis codes were gathered by the NHIS. The K-NHIS database contains information on demographics, medical treatments, procedures, prescription drugs, diagnostic codes, and hospital use. Survival status and cause of death were obtained from death certificates collected by Statistics Korea at the Ministry of Strategy and Finance of South Korea [20]. Use of the K-NHIS database was approved by the NHIS review committee. The Institutional Review Board of Samsung Medical Center approved the study and waived the requirement for informed consent because the K-NHIS data were de-identified (SMC 2022–03–028).

### Study population

From the K-NHIS database, we selected all patients aged 15–39 years diagnosed with cancer between 2006 and 2019, and four times their number in age- and sex-matched control samples. Among the 3,352,310 participants, those who had any cancer (*n* = 45,732) or CVD (*N* = 66,317) before January 1, 2006, were excluded. After these exclusions, 3,243,041 individuals were included in the sequential cohorts (eFigure 1 in the Supplement).

To reduce potential selection and immortal time biases, we employed a sequential nested cohort design aligned with the principles of target trial emulation [21, 22]. Although random assignment to NHL was not possible, this design allowed for a structured and time-updated selection of exposed and unexposed individuals under clearly defined eligibility criteria, analogous to the conceptual structure of a trial. Each individual was assessed for eligibility at 6-month intervals. When a participant was newly diagnosed with NHL, the corresponding time point was designated as baseline, and matched controls were selected from the same time-specific risk set using propensity scores based on the covariates measured at that time (eFigure 2 in the Supplement). Given the possibility of repeated inclusion of individuals across time-specific cohorts, we calculated robust standard errors clustered at the individual level to account for within-person correlations. This process continued through June 1, 2019, resulting in a total of *N* = 59,561,447 person-intervals (unique individuals *N* = 3,108,601). As our focus was on NHL, we selected all patients with NHL (*N* = 4553) and matched them to a control group in a 1:3 ratio (*N* = 13,636).

### Definition of AYA survivors of cancer

To define incident cancer, we used a special registration code in addition to the ICD-10 diagnosis code. The NHIS established a special co-payment reduction program to enhance health coverage and relieve the financial burden on patients. Once patients are registered in the system, they pay only 5% of the total medical costs incurred for cancer-related medical care. Because enrollment in this co-payment reduction program is indicated by a special co-payment reduction code for cancer (V193) and requires a medical certificate from a physician, the cancer diagnoses included in this study were considered sufficiently reliable. This method has been used in previous studies [23]. NHL was confirmed based on the presence of the C82–C85 or C96 codes. We further categorized it into five groups: diffuse large B-cell lymphoma (DLBCL) (ICD-10, C83.3), follicular lymphoma (ICD-10, C82), mature T/NK cell lymphoma (ICD-10, C84), anaplastic large cell lymphoma (ALCL) (ICD-10, C84.6, C84.7), and others (C96 or uncoded). We only included patients diagnosed with NHL who had undergone at least one relevant treatment, including chemotherapy, radiotherapy, or hematopoietic stem cell transplantation (HSCT), with both inpatient and outpatient claims considered. Treatment exposure was defined based on the presence of relevant procedure codes for these treatments in the NHIS claims database regardless of the number or timing of their occurrence (eTable 1 in the Supplement).

### Study outcomes

The primary outcomes of this study were major cardiovascular events, including myocardial infarction, cardiomyopathy, heart failure, ischemic stroke, and hemorrhagic stroke. Thrombotic events, including deep vein thrombosis (DVT) and pulmonary embolism (PE), were analyzed separately. Cardiovascular outcomes were identified based on diagnostic records according to ICD-10 codes for either outpatient visits or hospitalizations (eTable 1 in the Supplement**)**. Follow-up was defined as the date of NHL diagnosis (or the matched index date for controls) until CVD occurrence, death, or the end of follow-up (December 31, 2020).

### Covariates

For covariates, we included age, sex, comorbidities, income, and residential area at baseline. Comorbidities were defined using claims data prior to the index date: a medical claim based on ICD-10 codes and at least one relevant prescription during the 2 years before the index date. Hypertension (I10–I13 or I15 and antihypertensive medication), diabetes mellitus (E11–E14 and antidiabetic medication), and dyslipidemia (E78 and lipid-lowering medication) were included. The income data at the time of the first screening were obtained from an insurance eligibility database. Income levels were categorized into percentile groups (≤ 30 th, > 30 th– ≤ 70 th, and > 70 th percentiles). The residential areas at the time of the first screening examination were classified as metropolitan or rural. Metropolitan areas were defined as Seoul, six metropolitan cities, and 15 cities with populations > 500,000 that have been officially designated as municipal cities (https://www.mois.go.kr).

### Statistical analyses

Participants were then classified into two groups based on whether they had developed cancer. In this process, propensity score (PS) matching (1:3, caliper < 0.1) was performed using the variables of age, sex, income, residential area, and the presence of comorbidities at cohort entry. The standardized mean difference (SMD) between the cancer and control groups was estimated to compare the distribution of variables used for matching. The variables used for matching were updated on the basis of the first date of each subsequent cohort. The cumulative incidence of each outcome was estimated using the Kaplan–Meier method, and log-rank tests were applied to evaluate the differences between the groups. Hazard ratios (HR) with 95% confidence intervals (CI) were calculated for the incidence of the clinical outcomes using the Cox regression model. Covariates with an SMD > 0.1 between groups were adjusted for in the survival analyses. However, because treatment information was only available for the NHL group and did not apply to the control population, the treatment modality was not included as an adjustment variable. We examined the proportional hazard assumption using plots of the log(− log) survival function and Schoenfeld residuals.

In the sensitivity analysis, to account for competing risks due to mortality, we fitted a proportional sub-distribution hazard regression model [24] with death as the competing event. For the additional sensitivity analysis, we used a conventional cohort design without data replication. In this approach, individuals without prior cancer or CVD were followed up from January 2006 or from the time they reached 15 years of age. The NHL diagnosis was modeled as a time-varying exposure. To evaluate the potential influence of acute treatment-related effects, we conducted a landmark analysis excluding events that occurred within the first 2 years after cohort entry. Participants who developed CVD or died within the initial period were censored, and subsequent incident events were analyzed using Cox proportional hazards models. In the subgroup analysis, we analyzed the incidence of CVD according to the type of cancer treatment (chemotherapy only, radiation therapy only, both chemotherapy and radiation therapy, and HSCT). We also performed multivariable Cox regression analysis to identify the risk factors for CVD among patients with NHL.

All *P*-values were two-sided, and a *P*-value of < 0.05 was considered significant. Analyses were performed with the use of SAS® Visual Analytics (SAS Institute Inc., USA) and R 4.1.2 (R Foundation for Statistical Computing, Vienna, Austria).

## Results

**Fig. 1:**
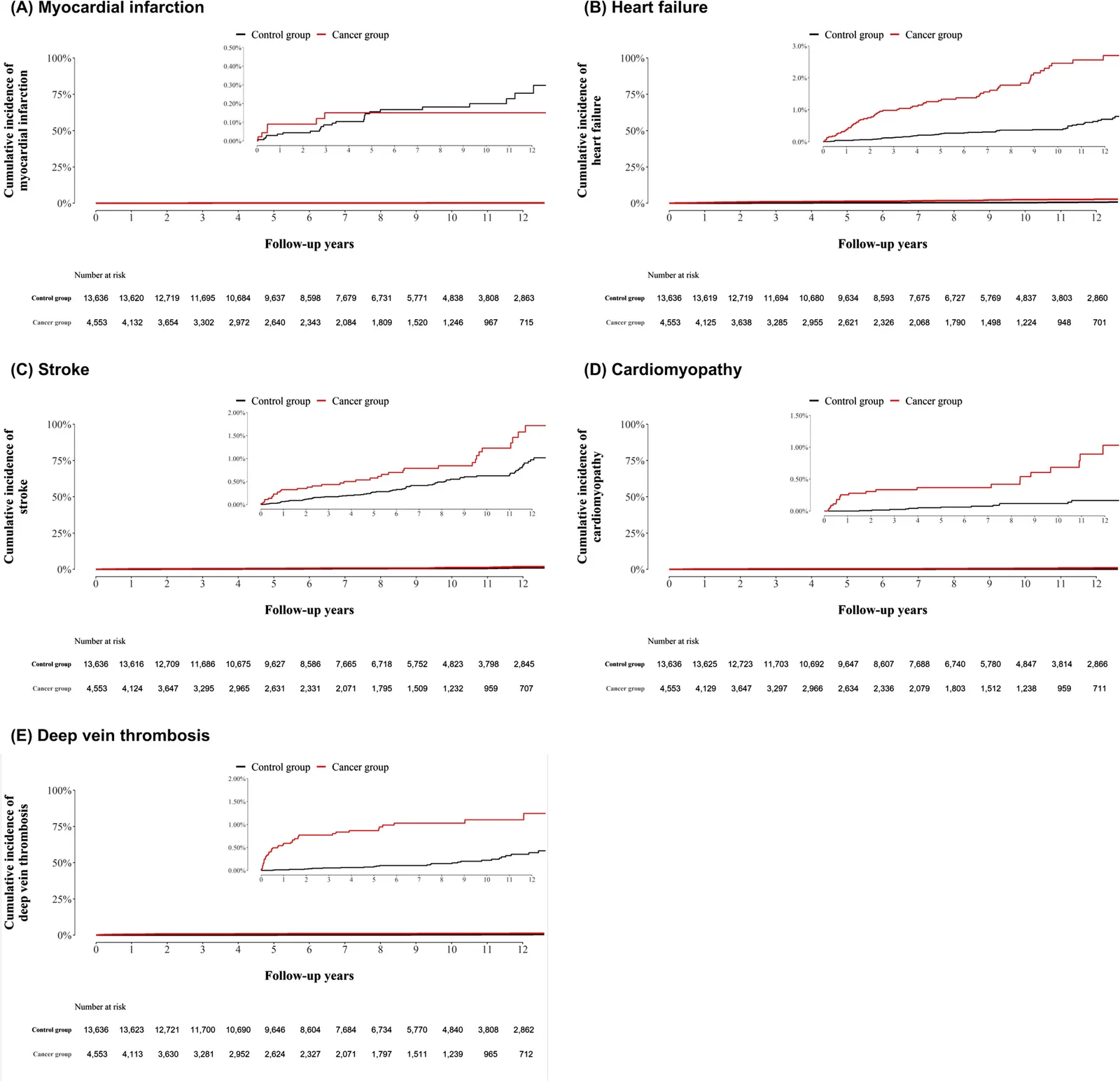
Kaplan–Meier curve for incidence of **A** myocardial infarction, **B** heart failure, **C** stroke, **D** cardiomyopathy, and **E** deep vein thrombosis

The mean age of the participants was 29.5 years, and 59% were men (Table 1). All standardized mean differences of the differences between the control and cancer groups were < 0.1. Among patients with NHL, the most frequent treatment was chemotherapy (56.3%), followed by chemotherapy and radiotherapy (20.5%). During follow-up (median, 6.5 years), 197 patients with NHL developed major cardiovascular events. Figure 1 shows the cumulative incidence of cardiovascular events among AYA survivors of NHL compared with matched controls. Compared with the control group, patients with NHL had a significantly higher risk of cardiomyopathy (HR 6.77; 95% CI 3.44–13.33), heart failure (HR 4.90; 95% CI 3.45–6.97), and hemorrhagic stroke (HR 3.14; 95% CI 1.75–5.65) (Table 2). These associations remained significant in competing risk analyses. No significant differences were observed for myocardial infarction (HR 0.87; 95% CI 0.35–2.12) or ischemic stroke (HR 1.30; 95% CI 0.77–2.20).

**Table 1:** Baseline characteristics

	Control	NHL	SMD
	*N* = 13,636	*N* = 4,553	
Age at cancer diagnosis, year (mean (SD))	29.5 (6.8)	29.4 (7.1)	− 0.010
Age group (%)			
15–19	1522 (11.2)	564 (12.4)	0.038
20–29	4490 (32.9)	1472 (32.3)	− 0.013
30–39	7624 (55.9)	2517 (55.3)	− 0.013
Sex (%)			− 0.004
Male	8118 (59.5)	2701 (59.3)	
Female	5518 (40.5)	1852 (40.7)	
Income (%)			
Medical aid	282 (2.1)	92 (2.0)	− 0.003
≤ 30 th	2895 (20.9)	944 (20.7)	− 0.005
31 st–70 th	5495 (40.3)	1838 (40.4)	0.001
> 70 th	4783 (35.1)	1603 (35.2)	0.003
Residential area, metropolitan (%)	9538 (69.9)	3184 (69.9)	0
Comorbidities			
Diabetes, yes (%)	444 (3.3)	159 (3.5)	0.013
Hypertension, yes (%)	804 (5.9)	269 (5.9)	0.001
Hyperlipidemia, yes (%)	995 (7.3)	335 (7.4)	0.002
Histologic subtypes (%)			
Diffuse large B-cell lymphoma (DLBCL)	-	1,218 (26.8)	
Follicular lymphoma (FL)	-	284 (6.2)	
Mature T/NK-cell lymphomas	-	395 (8.7)	
Anaplastic large cell lymphoma	-	112 (2.5)	
Others	-	2544 (55.9)	
Type of treatment (%)			
Radiotherapy only		474 (10.4)	
Chemotherapy only	-	2,562 (56.3)	
Chemotherapy + radiotherapy	-	934 (20.5)	
Chemotherapy + HSCT		392 (8.6)	
Chemotherapy + radiotherapy + HSCT		190 (4.2)	

*NHL* non-Hodgkin lymphoma, *HSCT* hematopoietic stem cell transplantation

**Table 2:** Hazard ratio (95% confidence intervals) for incident cardiovascular disease associated with non-Hodgkin lymphoma

	No of case (100 person-year)		HR (95% CI)*	SubHR (95% CI) †
	Control	NHL		
Myocardial infarction (I21–I22)	24 (0.0)	6 (0.0)	0.87 (0.35, 2.12)	0.75 (0.31, 1.84)
Cardiomyopathy (I42–I43, I23.5)	13 (0.0)	24 (0.1)	**6.77 (3.44, 13.33)**	**5.62 (2.86, 11.05)**
Heart failure (I50)	54 (0.1)	73 (0.3)	**4.90 (3.45, 6.97)**	**4.13 (2.91, 5.87)**
Ischemic stroke (I63–I64)	54 (0.1)	19 (0.1)	1.30 (0.77, 2.20)	1.07 (0.64, 1.81)
Hemorrhagic stroke (I60–I62)	24 (0.0)	21 (0.1)	**3.14 (1.75, 5.65)**	**2.66 (1.48, 4.77)**

^*^All covariates matched well

^†^Subhazard ratios for events were modeled with mortality as a competing risk

Statistically significant values are denoted in bold

In subgroup analyses stratified by treatment modality, patients who received only radiation therapy did not show a statistically significant increase in the risk of any individual cardiovascular event compared to the control group. In contrast, patients who underwent HSCT exhibited substantially higher risks, particularly for cardiomyopathy (HR 17.08; 95% CI 5.88–49.58) and heart failure (HR 8.03; 95% CI 4.16–15.48) (Table 3). Notably, in contrast to other cardiovascular outcomes, the risk of myocardial infarction increased markedly in individuals with two or more comorbid conditions compared with those without comorbidities (eTable 2 in the Supplement).

**Table 3:** Hazard ratio (95% confidence intervals) for incident cardiovascular disease by treatment

	No of case (100 person-year)	HR (95% CI)* comparing control	subHR (95% CI) † comparing control
Radiotherapy only			
Myocardial infarction (I21–I22)	0 (0)	-	-
Cardiomyopathy (I42–I43, I23.5)	1 (0)	3.63 (0.44, 30.16)	3.47 (0.41, 29.35)
Heart failure (I50)	4 (0.1)	1.73 (0.62, 4.80)	1.67 (0.61, 4.62)
Ischemic stroke (I63–I64)	1 (0)	0.65 (0.09, 4.78)	0.61 (0.08, 4.47)
Hemorrhagic stroke (I60–I62)	2 (0)	2.62 (0.61, 11.26)	2.30 (0.54, 9.84)
Chemotherapy only			
Myocardial infarction (I21–I22)	4 (0.0)	0.99 (0.34, 2.85)	0.91 (0.32, 2.61)
Cardiomyopathy (I42–I43, I23.5)	16 (0.1)	**7.68 (3.68, 16.00)**	**6.82 (3.28, 14.16)**
Heart failure (I50)	43 (0.3)	**5.10 (3.41, 7.61)**	**4.45 (2.98, 6.63)**
Ischemic stroke (I63–I64)	10 (0.1)	1.18 (0.60, 2.32)	1.03 (0.52, 2.03)
Hemorrhagic stroke (I60–I62)	11 (0.1)	**2.86 (1.40, 5.85)**	**2.53 (1.24, 5.14)**
Chemotherapy + radiotherapy			
Myocardial infarction (I21–I22)	1 (0)	0.81 (0.11, 6.04)	0.58 (0.08, 4.29)
Cardiomyopathy (I42–I43, I23.5)	2 (0)	**5.63 (1.16, 27.31)**	3.99 (0.84, 18.91)
Heart failure (I50)	14 (0.2)	**4.71 (2.62, 8.47)**	**3.40 (1.89, 6.09)**
Ischemic stroke (I63–I64)	6 (0.1)	**2.92 (1.24, 6.91)**	2.10 (0.89, 4.95)
Hemorrhagic stroke (I60–I62)	8 (0.1)	**7.02 (3.10, 15.89)**	**5.10 (2.27, 11.47)**
HSCT + chemotherapy ± radiotherapy			
Myocardial infarction (I21–I22)	1 (0.0)	1.65 (0.22, 12.26)	0.99 (0.13, 7.32)
Cardiomyopathy (I42–I43, I23.5)	5 (0.2)	**17.08 (5.88, 49.58)**	**9.25 (3.29, 26.04)**
Heart failure (I50)	11 (0.4)	**8.03 (4.16, 15.48)**	**5.04 (2.63, 9.63)**
Ischemic stroke (I63–I64)	2 (0.1)	1.64 (0.40, 6.79)	0.94 (0.23, 3.84)
Hemorrhagic stroke (I60–I62)	0 (0)	-	-

*HSCT* hematopoietic stem cell transplantation

^*^All covariates matched well

^†^Subhazard ratios for events were modeled with mortality as a competing risk

Statistically significant values are denoted in bold

In addition to major adverse cardiovascular events, we examined the incidence of thrombotic events, including DVT and PE, which are presented in eTable 3 in the Supplement. Compared with the control group, patients with NHL had significantly increased risks of both DVT (HR 5.05; 95% CI 3.15–8.12) and PE (HR 3.61; 95% CI 1.50–8.70). Similar to major cardiovascular events, the highest incidences of DVT and PE were observed in patients treated with HSCT (eTable 4 in the Supplement).

In the landmark analysis limited to events occurring after 2 years of follow-up, survivors of NHL continued to exhibit a higher risk of cardiomyopathy (HR 3.60; 95% CI 1.59–8.17) and heart failure (HR 3.56; 95% CI 2.34–5.42) compared to controls (eTable 5 in the Supplement). Sensitivity analysis using a conventional cohort design without data replication yielded similar results, reinforcing the robustness of the primary analysis (eTable 6 in the Supplement).

## Discussion

In this nationwide cohort study, AYA survivors of NHL demonstrated varying risks for different cardiovascular diseases. Notably, the risk of cardiomyopathy was more than six times higher, and the risk of heart failure, hemorrhagic stroke, DVT, and PE was more than three times higher. Although radiotherapy alone was not associated with the overall CVD risk, multimodal therapy was associated with an increased risk. This association remained significant, even after accounting for the competing risk of death.

To the best of our knowledge, this is the first study to characterize various cardiovascular outcomes in AYA survivors with NHL residing outside North America or Europe. Our findings of a significantly higher incidence of cardiomyopathy and heart failure are consistent with those of previous AYA studies. Kero et al. reported that childhood and AYA survivors of NHL had higher risks of various CVD subtypes, with the largest increases in the risk observed for cardiomyopathy/cardiac insufficiency (HR 9.2; 95% CI 5.8–14.7) [17]. Rugbjerg et al. documented an increased risk of cardiomyopathy (HR 6.49; 95% CI 4.26–9.91) and heart failure (HR 2.26; 95% CI 1.68–3.04) among AYA survivors of NHL [14]. Compared to studies on childhood cancer survivors, the cardiovascular risks observed in our AYA NHL cohort appeared to be of a lower magnitude [4, 7, 17]. This may reflect previous findings suggesting a greater vulnerability of younger children to the cardiotoxic effects of anthracycline [8, 25]. Additionally, the relatively shorter follow-up duration in our study may have limited the detection of very late-onset cardiovascular outcomes.

Interestingly, there was no significant difference in the risk of myocardial infarction or ischemic stroke between AYA survivors of NHL and controls. This result was comparable to another population-based study in which the risk of cerebral infarction was also not statistically significant (HR 1.51; 95% CI, 0.97–2.34), consistent with our results [14]. Similarly, a meta-analysis of four cohort studies (comprising two AYA, one children and AYA, and one adolescent and adult cohorts) assessing various CVD incidences in survivors of NHL found that coronary artery disease (CAD) was the only cardiovascular subtype that did not exhibit a statistically significant increased risk compared to the general population [26].A recent systematic review indicated that the most significant therapeutic factor associated with CAD development is radiation therapy involving exposure to the heart, with no significant association with chemotherapy [27]. According to a report from the European Association of Cardiovascular Imaging and the American Society of Echocardiography, CAD remains latent for at least 10 years after radiation exposure [28]. Moreover, the lower prevalence of traditional cardiac risk factors in AYA survivors at a relatively young age may contribute to the low incidence of CAD.

When examining the incidence curves, different latencies were observed across the cardiovascular outcomes. Cardiac conditions, such as cardiomyopathy and heart failure, showed a progressive increase in incidence over the entire follow-up period, suggesting long-term cumulative cardiotoxic effects. Arterial events, such as myocardial infarction, accumulate more gradually, which is consistent with the slower development of atherosclerosis. In contrast, venous thromboembolic events, DVT, and PE tended to occur earlier, suggesting a closer association with active cancer or acute treatment-related effects rather than delayed toxicity. Patients with cancer are at an increased risk of thromboembolism owing to multiple contributing factors, including the use of central venous catheters (CVCs) and exposure to certain chemotherapeutic agents. Catheter-related thrombosis is particularly common in children and AYA patients, given the frequent use of CVCs in these populations [29, 30]. In addition, malignancy induces a hypercoagulable state through elevated procoagulant factors, impaired fibrinolysis, stasis from immobility or increased blood viscosity, and endothelial injury, all of which contribute to a heightened risk of thrombosis [31, 32].

In subgroup analyses stratified by treatment modality, an elevated CVD risk was observed among patients who received chemotherapy alone, combined chemotherapy and radiotherapy, or intensive multimodal therapy including HSCT, whereas those treated with radiotherapy alone did not show a significant increase in risk. This may reflect advances in contemporary radiation techniques, such as reduced cardiac exposure through refined field planning and protocol optimization [33]. Alternatively, radiation-associated cardiotoxicity may have a longer latency period, potentially extending beyond the duration of our follow-up. Among the treatment groups, the patients who underwent HSCT had the highest risk of CVD. Although this may indicate a potential association with HSCT [34], it is more likely that the increased risk reflects the intensive treatment burden and underlying disease severity in patients who undergo HSCT, particularly those with relapsed or refractory aggressive NHL [35–37]. Due to the limitations of claims data, we were unable to obtain detailed treatment information, including transplant type and conditioning regimens. Therefore, further research using comprehensive clinical data is required to clarify the contributors to cardiovascular risk in this population.

The development of CVD in cancer survivors involves multiple factors, including direct treatment-related risks, preexisting and comorbid conditions, and changes in behavioral habits [38]. It is probable that AYA survivors have undetected subclinical CVD, which can be further exacerbated by exposure to anticancer therapies and alterations in their health behaviors. Moreover, cancer and CVD share multiple risk factors, such as smoking, physical inactivity [39], and inflammation [40]. A previous study reported that AYA survivors with at least one additional CVD risk factor had a 1.8- to 3.2-fold increased risk of developing CVD [41]. In our study, the risk of myocardial infarction was markedly increased in patients with multiple comorbidities, highlighting the need for comprehensive risk management.

This study has several strengths. AYA survivors of NHL in this cohort were diagnosed and treated in a fairly recent era of improved cancer care, thus enhancing the relevance of our findings to current clinical practice. Additionally, this study used the Korean National Health Insurance System, a comprehensive single-payer database that ensures universal coverage and captures nearly all nationwide inpatient and outpatient encounters. This enabled consistent healthcare access among both AYA survivors and the control group, minimizing the potential bias from differential medical service access. Nonetheless, our study has some limitations. First, the use of claims data limited access to certain clinical details, including disease stage at diagnosis and information on relapses. Second, detailed treatment-related data, such as chemotherapy regimens and dosages, radiation type and intensity, and conditioning regimens for HSCT, were unavailable. Although treatment exposure was identified using procedure codes with high specificity for the actual receipt of therapy, non-reimbursed treatments may not have been captured, potentially leading to an underestimation of exposure in some patients. Third, the definitions of exposures and outcomes were based on established operational criteria used in prior NHIS-based studies; however, some misclassifications may have occurred and affected the observed risk estimates. Nonetheless, given our focus on comparing cardiovascular risk in AYA survivors of NHL with that in the general population, the use of a comprehensive claims database with standardized coding remains a strength. Fourth, although we assessed multiple cardiovascular outcomes, they were pre-specified based on clinical relevance. We did not apply formal adjustments for multiple comparisons; therefore, the results should be interpreted with caution, particularly for outcomes with small event counts. Finally, although our models were adjusted for various potential confounders, unmeasured confounders, such as genetic factors, family history, and behavioral factors, may have influenced the results.

In conclusion, our cohort of more than 4500 AYA survivors of NHL had a higher risk of developing CVD, including cardiomyopathy, heart failure, hemorrhagic stroke, DVT, and PE, than the general population. Our study highlights the importance of monitoring cardiovascular health in high-risk AYA survivors of NHL who receive combined therapies, including chemotherapy with radiation, HSCT, or multiple cardiovascular risk factors. Considering the limited information available on AYA survivors, our data will be valuable for the future development of evidence-based guidelines for late effects screening and care tailored to this age group.

## Supplementary Information

Below is the link to the electronic supplementary material.

Supplementary file1 (DOCX 697 KB)

## Data Availability

No datasets were generated or analysed during the current study.
