# Supplementary material for: Cardiovascular disease in adolescent and young adult survivors of non-Hodgkin lymphoma in Korea
Source: J Cancer Surviv. 2025 May 16;20(Suppl 1):337–46. doi: 10.1007/s11764-025-01816-2 (PMC13676521; doi:10.1007/s11764-025-01816-2)
Supplement: Supplementary file 1 [file 11764_2025_1816_MOESM1_ESM.docx]

**Supplemental Online Content**

**eTable 1.** Definition of outcomes

**eTable** **2**. Risk factors of cardiovascular disease in NHL

**eTable 3.** Hazard ratio (95% confidence intervals) for deep vein thrombosis and pulmonary embolism associated with Non-Hodgkin Lymphoma

**eTable** **4**. Hazard ratio (95% confidence intervals) for deep vein thrombosis and pulmonary embolism by treatment

**eTable** **5**. Hazard ratio (95% confidence intervals) for cardiovascular outcomes associated with Non-Hodgkin Lymphoma beyond 2 years from diagnosis

**eTable 6.** Hazard ratio (95% confidence intervals) for incident cardiovascular disease using single cohort with Non-Hodgkin Lymphoma as time varying analysis

**eFigure 1.** Flow chart

**eFigure 2.** Schematic diagram of cohort construction

**eTable 1. Definition of outcomes**

| **Outcome** | **ICD 10 code or**  **Procedure code** | **Diagnostic definition** |
| --- | --- | --- |
| **Cancer therapy** |  |  |
| Chemotherapy | ATC code: L01A – L01F, L01X |  |
| Radiation therapy | HD010 - HD023,  HD031 - HD033,  HD040, HD041,  HD051 - HD061,  HD071 - HD073,  HD080 - HD089,  HD091- HD093,  HD110 - HD115,  HD121, HD150, HD160,  HD211, HD212,  HD410 - HD420,  HD441, HX401-HY405,  HZ271 |  |
| Hematopoietic stem cell transplantation | X5131-X5136, X5051 |  |
| **Cardiovascular disease** |  |  |
| Myocardial infraction | I21-I22 (Primary and 1^st^ sub) | Admission or outpatient department >= 2 |
| Cardiomyopathy | I42-I43, I23.5 | Admission or outpatient department >= 2 |
| Heart failure | I50 (Primary and 1^st^ sub) | Admission or outpatient department >= 2 |
| Ischemic stroke | I63-I64 (Primary and 1^st^ sub) | Admission or outpatient department >= 2 |
| Hemorrhagic stroke | I60-I62 (Primary and 1^st^ sub) | Admission or outpatient department >= 2 |
| Deep venous thromboembolism | I80.1-I80.3 | Admission or outpatient department >= 2 |
| Pulmonary embolism | I26 | Admission or outpatient department >= 2 |

**eTable 2. Risk factors of cardiovascular disease in NHL**

|  | **Myocardial infarction HR (95% CI)** | **Cardiomyopathy HR (95% CI)** | **Heart failure HR (95% CI)** | **Ischemic stroke HR (95% CI)** | **Hemorrhagic stroke HR (95% CI)** |
| --- | --- | --- | --- | --- | --- |
| **Age at diagnosis (years)** | 1.05 (0.92, 1.21) | 1.00 (0.95, 1.06) | 0.99 (0.96, 1.02) | 1.07 (0.99, 1.16) | 1.02 (0.96, 1.09) |
| **Age at cancer diagnosis (years)** |  |  |  |  |  |
| 15–19 | *Reference* | *Reference* | *Reference* | *Reference* | *Reference* |
| 20–29 | - | 0.96 (0.28, 3.27) | 0.92 (0.44, 1.97) | 1.52 (0.13, 13.77) | 1.85 (0.39, 8.92) |
| 30–39 | - | 0.93 (0.28, 3.05) | 0.92 (0.46, 1.83) | 3.25 (0.42, 25.28) | 1.47 (0.32, 6.85) |
| **Sex, Female** | 0.75 (0.14, 4.12) | 1.18 (0.52, 2.69) | 1.30 (0.82, 2.07) | 0.84 (0.33, 2.14) | 0.72 (0.29, 1.79) |
| **Income** |  |  |  |  |  |
| Medical Aid | - | **12.45 (3.30, 47.03)** | 2.88 (0.98, 8.45) | - | 3.67 (0.43, 31.36) |
| ≤30^th^ | 0.70 (0.07, 6.82) | 1.53 (0.46, 5.04) | 0.87 (0.44, 174) | 1.45 (0.46, 4.59) | 0.66 (0.13, 3.27) |
| 31^st^–70^th^ | *Reference* | *Reference* | *Reference* | *Reference* | *Reference* |
| >70^th^ | 0.73 (0.12, 4.40) | 1.68 (0.50, 4.76) | 1.43 (0.84, 2.42) | 1.15 (0.40, 3.29) | 2.31 (0.86, 6.18) |
| **Residential area, Metropolitan** | 2.02 (0.23, 17.45) | 0.77 (0.33, 1.81) | 0.62 (0.38, 0.99) | 0.64 (0.25, 1.64) | 0.95 (0.37, 2.47) |
| **Type of treatment** |  |  |  |  |  |
| Radiotherapy only | *Reference* | *Reference* | *Reference* | *Reference* | *Reference* |
| Chemotherapy only | - | 3.48 (0.45, 26.70) | 2.46 (0.88, 6.92) | 2.58 (0.33, 20.35) | 1.17 (0.26, 5.39) |
| Chemotherapy + Radiotherapy | - | 1.29 (0.12, 14.38) | 2.50 (0.83, 7.59) | 4.67 (0.56, 39.29) | 2.55 (0.53, 12.16) |
| HSCT + Chemotherapy ± Radiotherapy | - | 6.59 (0.75, 57.95) | **3.65 (1.15, 11.60)** | 3.33 (0.30, 37.50) | - |
| **Comorbidities** |  |  |  |  |  |
| Diabetes, yes | 2.68 (0.24, 29.90) | - | 1.37 (0.47, 3.97) | 1.08 (0.13, 8.93) | - |
| Hypertension, yes | 5.98 (0.88, 40.58) | 2.62 (0.70, 9.79) | 1.91 (0.82, 4.44) | 1.72 (0.36, 8.31) | 1.97 (0.43, 8.93) |
| Hyperlipidemia, yes | 0.97 (0.09, 10.81) | 1.55 (0.41, 5.87) | 1.10 (0.45, 2.69) | 1.50 (0.31, 7.15) | 1.30 (0.29, 5.90) |
| **Comorbidities** |  |  |  |  |  |
| 0 | *Reference* | *Reference* | *Reference* | *Reference* | *Reference* |
| 1 | - | 1.50 (0.20, 11.57) | 1.65 (0.84, 3.23) | 0.56 (0.07, 4.22) | 0.89 (0.20, 3.85) |
| 2+ | **13.80 (2.53, 75.40)** | 1.92 (0.64, 5.75) | 1.80 (0.56, 5.81) | 3.88 (0.85, 17.73) | 1.65 (0.21, 12.77) |

*Adjusted for the all covariates in the tables

**eTable 3. Hazard ratio (95% confidence intervals) for deep vein thrombosis and pulmonary embolism associated with Non-Hodgkin Lymphoma**

|  | **No of case**  **(100 person-year)** | | **HR (95% CI)*** | **SubHR (95% CI)** † |
| --- | --- | --- | --- | --- |
|  | **Control** | **NHL** |  |  |
| **Deep vein thrombosis (I80.1**–**I80.3)** | 29 (0.0) | 42 (0.1) | **5.05 (3.15, 8.12)** | **4.41 (2.75, 7.06)** |
| **Pulmonary embolism (I26)** | 10 (0.0) | 10 (0.0) | **3.61 (1.50, 8.70)** | **3.07 (1.29, 11.30)** |

* All covariates matched well

† Subhazard ratios for events were modeled with mortality as a competing risk.

**eTable 4. Hazard ratio (95% confidence intervals) for deep vein thrombosis and pulmonary embolism by treatment**

|  | **No of case**  **(100 person-year)** | **HR (95% CI)***  **comparing control** | **subHR (95% CI)** † **comparing control** |
| --- | --- | --- | --- |
| **Radiotherapy only** |  |  |  |
| **Deep vein thrombosis (I80.1**–**I80.3)** | 2 (0) | 1.79 (0.42, 7.58) | 1.64 (0.39, 6.94) |
| **Pulmonary embolism (I26)** | 0 (0) | - | - |
| **Chemotherapy only** |  |  |  |
| **Deep vein thrombosis (I80.1**–**I80.3)** | 21 (0.1) | **4.54 (2.58, 7.97)** | **4.02 (2.31, 7.01)** |
| **Pulmonary embolism (I26)** | 4 (0.0) | 2.62 (0.82, 8.40) | 2.27 (0.72, 7.10) |
| **Chemotherapy + Radiotherapy** |  |  |  |
| **Deep vein thrombosis (I80.1**–**I80.3)** | 9 (0.2) | **5.05 (2.39, 10.66)** | **4.19 (1.98, 8.86)** |
| **Pulmonary embolism (I26)** | 3 (0.1) | **4.50 (1.25, 16.22)** | **3.76 (1.04, 13.57)** |
| **Hematopoietic stem cell transplantation** |  |  |  |
| **Deep vein thrombosis (I80.1**–**I80.3)** | 10 (0.3) | **13.84 (6.59, 29.03)** | **8.87 (4.34, 18.10)** |
| **Pulmonary embolism (I26)** | 3 (0.1) | **15.27 (3.87, 60.25)** | **8.44 (2.40, 29.71)** |

* All covariates matched well

† Subhazard ratios for events were modeled with mortality as a competing risk.

**eTable 5.** Hazard ratio (95% confidence intervals) for cardiovascular outcomes associated with Non-Hodgkin Lymphoma beyond 2 years from diagnosis

|  | **HR (95% CI)*** | **SubHR (95% CI)**† |
| --- | --- | --- |
|  |  |  |
| **Myocardial infarction (I21**–**I22)** | 0.40 (0.09, 1.72) | 0.39 (0.09, 1.68) |
| **Cardiomyopathy (I42**–**I43, I23.5)** | **3.60 (1.59, 8.17)** | **3.29 (1.44, 7.50)** |
| **Heart failure (I50)** | **3.56 (2.34, 5.42)** | **3.36 (2.20, 5.11)** |
| **Ischemic stroke (I63**–**I64)** | 1.14 (0.62, 2.13) | 1.06 (0.57, 1.96) |
| **Hemorrhagic stroke (I60**–**I62)** | 2.10 (0.97, 4.56) | 1.98 (0.92, 4.29) |

* All covariates matched well

† Subhazard ratios for events were modeled with mortality as a competing risk.

**eTable 6.** Hazard ratio (95% confidence intervals) for incident cardiovascular disease using single cohort with Non-Hodgkin Lymphoma as time varying analysis

|  | **HR (95% CI)*** |
| --- | --- |
|  |  |
| **Myocardial infarction (I21**–**I22)** | 0.92 (0.41, 2.06) |
| **Cardiomyopathy (I42**–**I43, I23.5)** | **5.83 (3.84, 8.86)** |
| **Heart failure (I50)** | **4.15 (3.29, 5.23)** |
| **Ischemic stroke (I63**–**I64)** | 1.25 (0.80, 1.96) |
| **Hemorrhagic stroke (I60**–**I62)** | **2.14 (1.36, 3.37)** |

**eFigure 1. Flow chart**

**eFigure 2.** Schematic diagram of cohort construction

Each participant was assessed for eligibility at 6-month intervals. Once diagnosed with NHL, the corresponding time point became the baseline for that individual, and matched controls were selected from the same time-specific risk set using propensity score matching.
